# Supplementary material for: Tobacco BY2 cells expressing recombinant cardosin B as an alternative for production of active milk clotting enzymes
Source: Sci Rep. 2021 Jul 14;11:14501. doi: 10.1038/s41598-021-93882-6 (PMC8280186; doi:10.1038/s41598-021-93882-6)
Supplement: Supplementary file 1 — Supplementary Figures. [file 41598_2021_93882_MOESM1_ESM.pdf]

SUPPLEMENTARY MATERIAL

Tobacco BY2 cells expressing recombinant cardosin B as an alternative for production of active milk clotting enzymes

André Folgado & Rita Abranches

Plant Cell Biology Laboratory, Instituto de Tecnologia Química e Biológica António Xavier, ITQB NOVA, Universidade Nova de Lisboa, 2780-157 Oeiras, Portugal

Figure S1

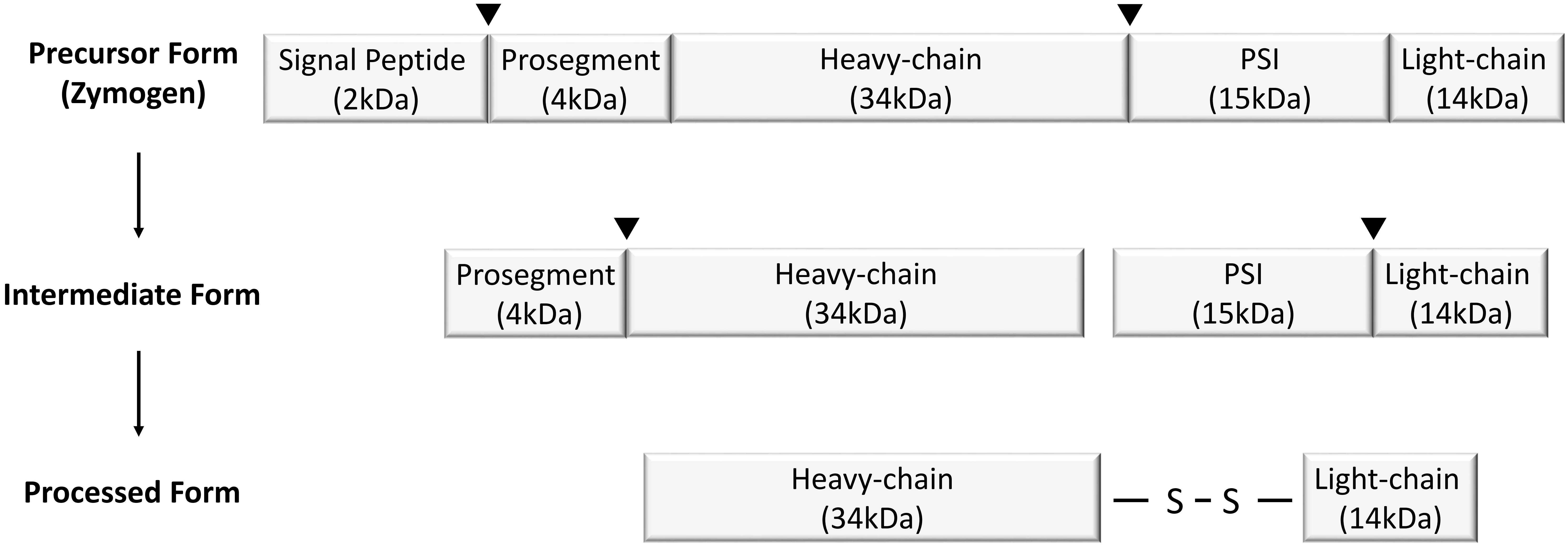

Figure S1. Schematic representation of cardosin B processing steps. Arrowheads indicate cleavage site during processing. PSI – Plant Specific Insert. S-S indicates disulfide bond between heavy- and light-chains.

Figure S2

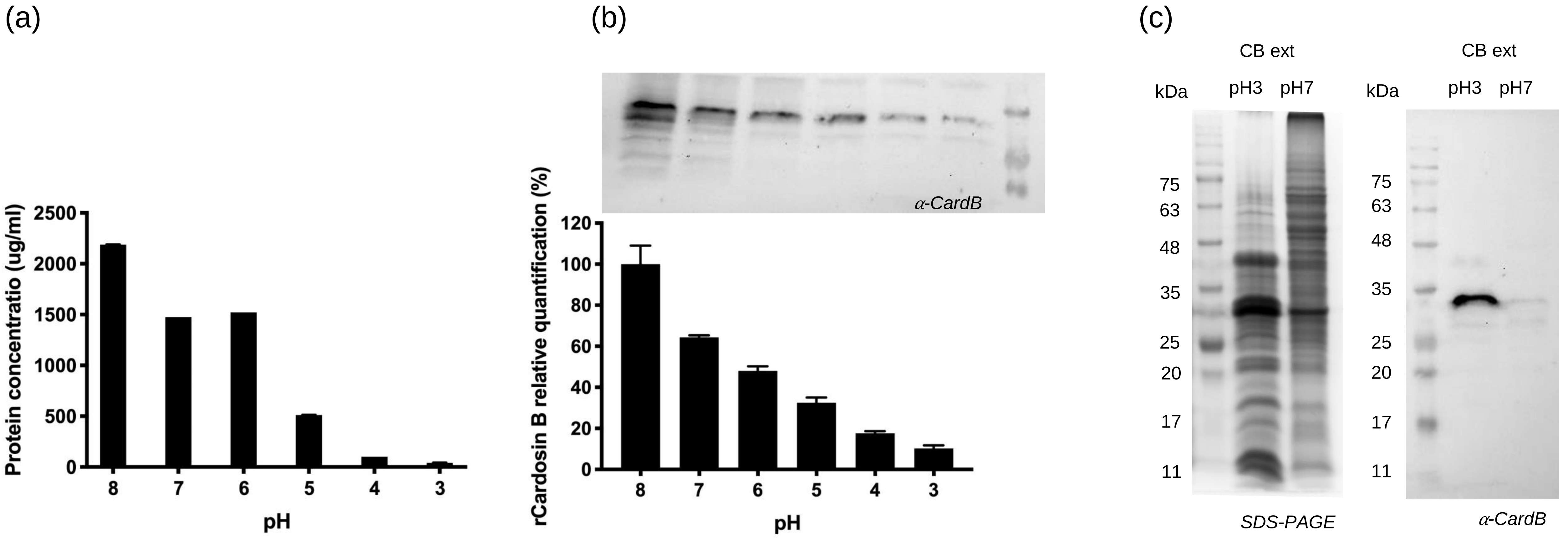

Figure S2. Optimization of cellular extractions. (a) Effect of pH on cell extract protein concentration (b) Effect of pH on cardosin B extraction with corresponding western blotting of the protein extracts at various pH values. The values are the mean of three western blot replicates (c) SDS-PAGE and western blot analysis of cell extracts at distinct pH values normalized to protein concentration.

Figure S3

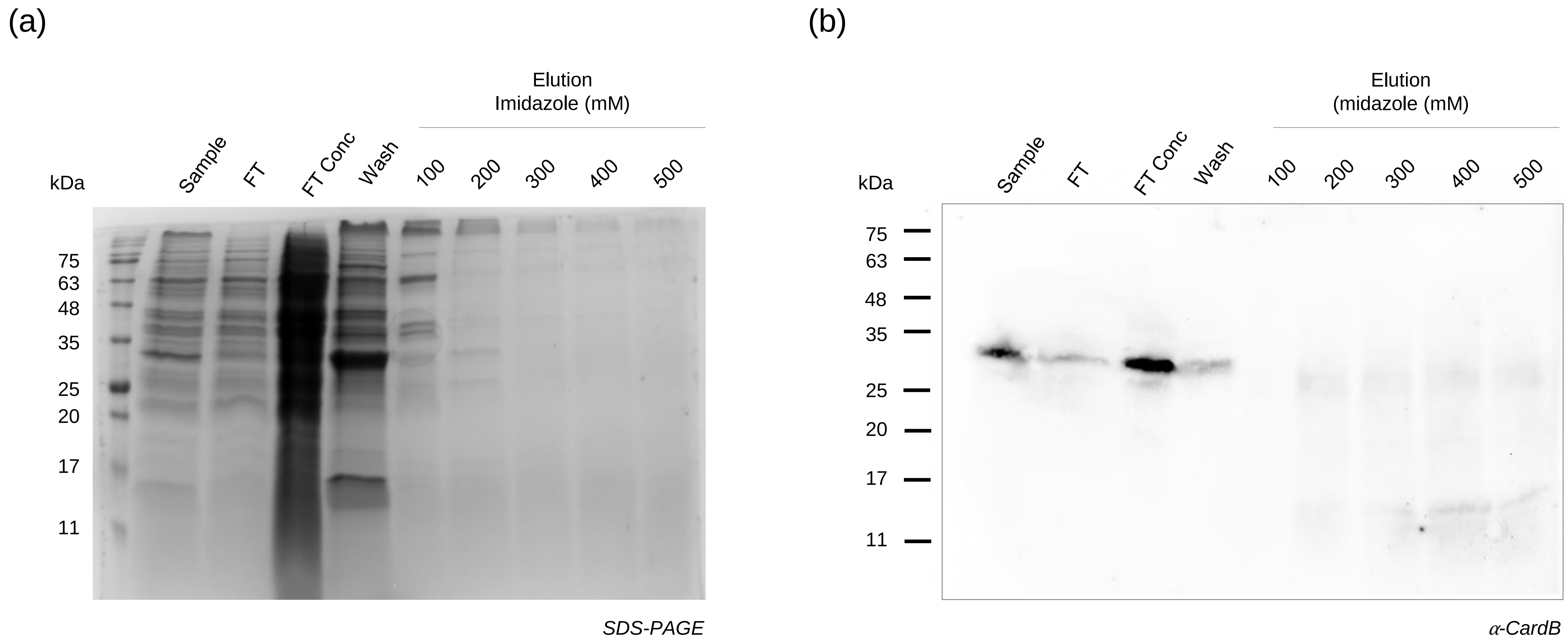

Figure S3. Purification of cardosin B by immobilized metal affinity using a His Trap™ column. (a) SDS-PAGE analysis (b) Western blot analysis (FT = flow through).

Figure S4

34kDa

MGTPIKASLLALFLFFLLSPTAFSVSNGGLLRVGLKKRKVDRLDQLRAHGVHMLGNARKDFGFRRTLSDSGSGIVALTNDRDTAYYGEIGIGTPPQNFAVIFDTGSSDLWVPSTKCDTSLACVIHPRYDSGDSSTYKGNGTTASIQYGTGAIVGFYSQDSVEVGDLVVEHQDFIETTEEDDTVFLKSEFDGILGLGFQEISAGKA
VPVWYNMVNQGLVEEAVFSFWLNRNVDEEEGGELVFGGVDPNHFRGNHTYVPVTRKGYWQFEMGDVLIGDKSSGFCAGGCAAIADSGTSFFAGPTAIITQINQAIGAKGVLNQQCKTLVGQYGKNMIQMLTSEVQPDKICSHMKLCTFDGAHDVRSMIESVVDKNNDKSSGGEICTFCEMALVRMQNEIKRNETEDNIINHVNEV
CDQLPTSSAESIVDCNGISSMPNIAFTIGSKLFEVTPEQYIYKVGEGEAATCISGFTALDIMSPQGPIWILGDMFMGPYHTVFDYGKLRVGFAEAV

±30kDa

MGTPIKASLLALFLFFLLSPTAFSVSNGGLLRVGLKKRKVDRLDQLRAHGVHMLGNARKDFGFRRTLSDSGSGIVALTNDRDTAYYGEIGIGTPPQNFAVIFDTGSSDLWVPSTKCDTSLACVIHPRYDSGDSSTYKGNGTTASIQYGTGAIVGFYSQDSVEVGDLVVEHQDFIETTEEDDTVFLKSEFDGILGLGFQEISAGKA
VPVWYNMVNQGLVEEAVFSFWLNRNVDEEEGGELVFGGVDPNHFRGNHTYVPVTRKGYWQFEMGDVLIGDKSSGFCAGGCAAIADSGTSFFAGPTAIITQINQAIGAKGVLNQQCKTLVGQYGKNMIQMLTSEVQPDKICSHMKLCTFDGAHDVRSMIESVVDKNNDKSSGGEICTFCEMALVRMQNEIKRNETEDNIINHVNEV
CDQLPTSSAESIVDCNGISSMPNIAFTIGSKLFEVTPEQYIYKVGEGEAATCISGFTALDIMSPQGPIWILGDMFMGPYHTVFDYGKLRVGFAEAV

10kDa

SAESIVDCNGISSMPNIAFTIGSKLFEVTPEQYIYKVGEGEAATCISGFTALDIMSPQGPIWILGDMFMGPYHTVFDYGKLRVGFAEAVVDGGGGSAAAHHHHHH

Figure S4. Analysis of purified cardosin B from BY2 cells by Triple TOF-MS. Sequences in green were identified with a value of confidence > 95%, sequences in yellow were identified with a value of confidence between 95% and 50% and sequences in red were identified with a value of confidence < 50%

Figure S5

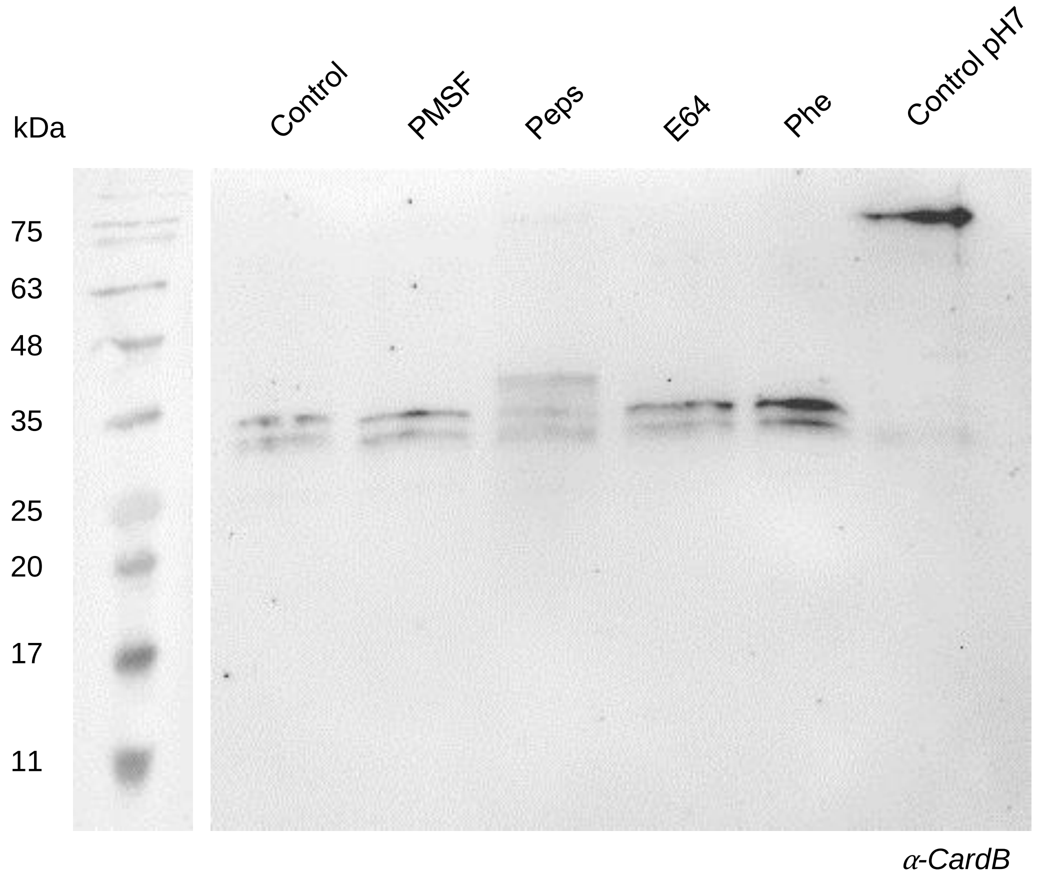

Figure S5. Western blot analysis of activation assay of unprocessed cardosin B with protease class specific inhibitors. Peps, pepstatin A (Aspartic protease inhibitor); PMSF, phenylmethylsulfonyl fluoride (Serine protease inhibitor); Phe, phenanthroline (Metalloprotease inhibitor); E64 (Cysteine protease inhibitor).

Figure S6

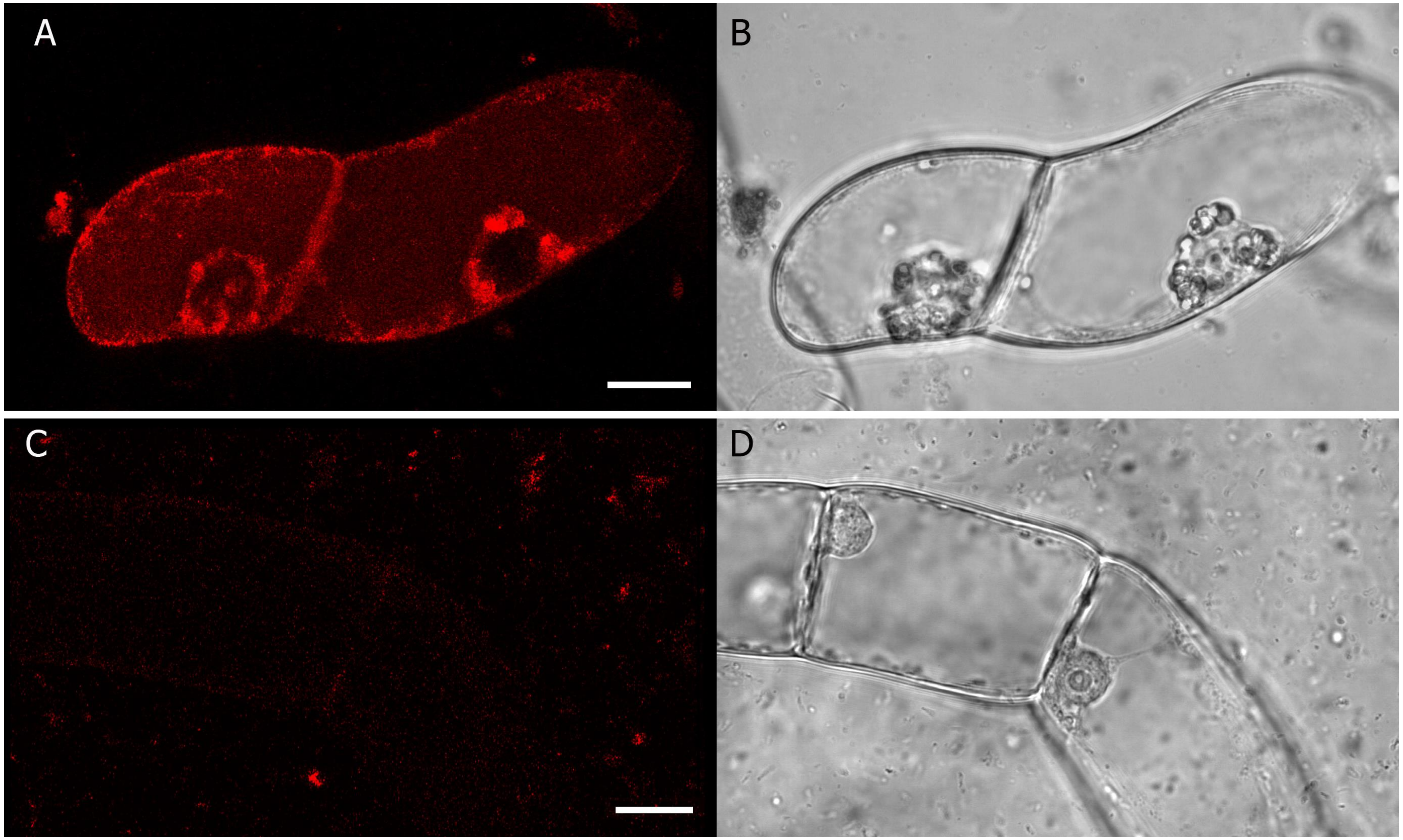

Figure S6. Confocal images of BY2 cells from line CardB-dsRed (upper panel) and wild type (lower panel). A and C dsRed; B and D Brightfield

CardB-dsRed cells clearly show vesicles around the nucleus. Red fluorescence signal is visible in these vesicles and also in the volume of the cell likely corresponding to the vacuole, as opposed to wild type . Wild type image does not show any red fluorescence (levels have been enhanced so that some background signal is visible). Bar 15 µM.

Figure S7

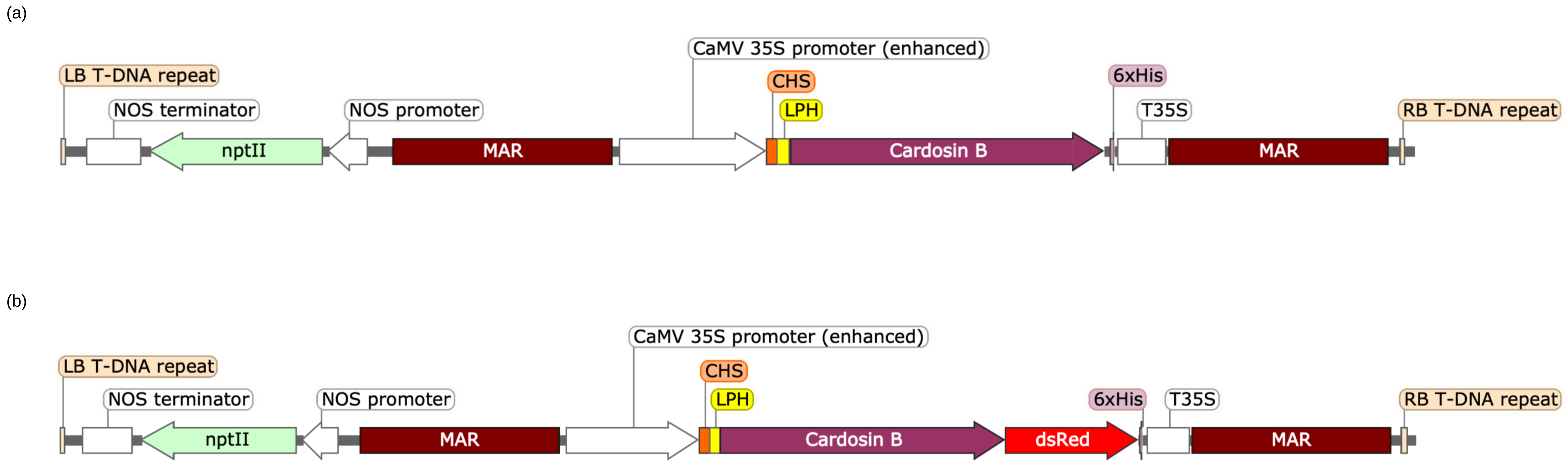

Figure S7. Schematic representation of the T-DNA cassette used in each vector. (a) pTRA-CardosinB (b) pTRA-CardosinB-dsRed. LB, Left border; nptII, kanamycin resistance marker; MAR, matrix attachment region; CaMV 35SS promoter, cauliflower mosaic virus 35S promoter; CHS, 5´UTR from chalcone synthase; LPH, murine signal peptide; 6xHis, six histidine tag; T35S, 35S terminator; RB, Right border.
